# Probing the intermolecular interactions of PPARγ-LBD with polyunsaturated fatty acids and their anti-inflammatory metabolites to infer most potential binding moieties

**DOI:** 10.1186/s12944-016-0404-3

**Published:** 2017-01-21

**Authors:** Shalini Muralikumar, Umashankar Vetrivel, Angayarkanni Narayanasamy, Undurti N. Das

**Affiliations:** 1 Centre for Bioinformatics, Kamalnayan Bajaj Institute for Research in Vision and Ophthalmology, Vision Research Foundation, Sankara Nethralaya, Chennai, 600 006 Tamil Nadu India; 2 Department of Biochemistry and Cell Biology, Kamalnayan Bajaj Institute for Research in Vision and Ophthalmology, Vision Research Foundation, Sankara Nethralaya, Chennai, 600 006 Tamil Nadu India; 3 UND Life Sciences, 2020 S 360th St, # K202, Federal Way, WA 98003 USA; 4 BioScience Research Centre, GVP College of Engineering, Visakhapatnam, 530048 India

**Keywords:** PPARγ, PUFA, Bioactive lipids, Docking, Helix12

## Abstract

**Background:**

PPARγ is an isoform of peroxisome proliferator-activated receptor (PPAR) belonging to a super family of nuclear receptors. PPARγ receptor is found to play a crucial role in the modulation of lipid and glucose homeostasis. Its commotion has been reported to play a significant role in a broad spectrum of diseases such as type 2 diabetes mellitus, inflammatory diseases, Alzheimer’s disease, and in some cancers. Hence, PPARγ is an important therapeutic target. Polyunsaturated fatty acids (PUFAs) and their metabolites (henceforth referred to as bioactive lipids) are known to function as agonists of PPARγ. However, agonistic binding modes and affinity of these ligands to PPARγ are yet to be deciphered.

**Methods:**

In this study, we performed a comparative molecular docking, binding free energy calculation and molecular dynamics simulation to infer and rank bioactive lipids based on the binding affinities with the ligand binding domain (LBD) of PPARγ.

**Results:**

The results inferred affinity in the order of resolvin E1 > neuroprotectin D1 > hydroxy-linoleic acid > docosahexaenoic acid > lipoxin A4 > gamma-linolenic acid, arachidonic acid > alpha-linolenic acid > eicosapentaenoic acid > linoleic acid. Of all the bioactive lipids studied, resolvin E1, neuroprotectin D1 and hydroxy-linoleic acid showed significant affinity comparable to proven PPARγ agonist namely, rosiglitazone, in terms of Glide XP docking score, H-bond formation with the key residues, binding free energy and stable complex formation with LBD favouring co-activator binding, as inferred through Molecular Dynamics trajectory analysis.

**Conclusion:**

Hence, these three bioactive lipids (resolvin E1, neuroprotectin D1 and hydroxy-linoleic acid) may be favourably considered as ideal drug candidates in therapeutic modulation of clinical conditions such as type 2 DM, Alzheimer’s disease and other instances where PPARγ is a key player.

## Background

Peroxisome proliferator-activated receptor (PPAR) comprises of three isoforms, α, β and γ belonging to a super family of nuclear receptors [1]. PPARs are ligand- activated transcription factors that regulate genes playing a vital role over a broad spectrum of physiological and pathological conditions [2]. PPAR receptors are expressed by various tissues including muscles, hepatocytes, adipocytes and endothelial cells. Though the three isoforms of PPAR (α, β and γ) share high level of sequence and structural similarity they are distinct in terms of expression and tissue distribution [3]. Molecular 3D structure of PPAR constitutes DNA binding domain at the N-terminus and ligand binding domain (LBD) at the C-terminus. PPAR’s interaction with its agonist leads to heterodimer formation with retinoid X receptor (RXR) [4]. PPAR-RXR heterodimer gets bounded at peroxisome proliferator response elements (PPREs) occupying the promoter region of target specific genes. Further, this process leads to recruitment of various transcriptional cofactors involved in the initiation of transcription process, thereby, triggering expression of several genes involved in diverse physiological and pathological processes [5–7]. Each PPAR subtype plays a unique physiological role in different tissues; however, all the three isoforms are well known to be involved in lipid and glucose homeostasis [8]. Of all the three isoforms, PPAR α and γ are most extensively studied when compared to PPAR β.

PPARα is predominantly expressed in tissues involved in metabolic activities of various tissues including muscles, heart, liver, intestine and brown adipose cells. PPARα activation leads to a decrease in lipid levels. PPARα receptor functions as a lipid sensor and helps in controlling energy combustion [9–12]. PPARγ is widely expressed in adipocytes, thereby, playing a crucial role in adipogenesis, lipid synthesis and in maintaining energy balance. PPARγ activation improves insulin sensitivity. In addition, PPARγ is expressed in spleen, large intestine, white and brown adipose tissues that may account for the involvement of these tissues in the pathobiology of type 2 Diabetes Mellitus and metabolic syndrome [10] [13, 14]. PPARβ is abundantly expressed in liver and abdominal adipose tissues by which it regulates blood cholesterol, glucose levels and influences fatty acid oxidation in cardiac and skeletal muscles [15, 16].

In the present study, we focussed on PPARγ receptor, as it plays a critical extensive role over broad spectrum of diseases such as type 2 diabetes mellitus, inflammatory diseases, Alzheimer’s disease, and in some cancers [17–23]. PPARγ comprises a Y-shaped ligand binding domain (LBD), which is segmented into three arms, arm I, arm II and arm III. Arm I is extended towards helix12 (H12), known to be polar and widely conserved across the PPAR isoform [24–26]. It also harbors transcription activation function-2 (AF-2) at C-terminal region and is held in its active conformation by the hydrogen bonding network with Arm I favouring ligand binding [27, 28]. Arm II and Arm III are found to be less conserved compared to Arm I and are hydrophobic in nature [29]. It has been proposed that diverse ligands bind to PPARγ with different binding modes to LBD with most of the ligand binding scenarios displaying a hydrophilic interaction with Arm I region and hydrophobic interactions with either arm II or arm III regions [30].

It has been proposed that full agonists of PPARγ reside over a large area of LBD of PPARγ with a U-shaped conformation and ideally comprising a polar head and a hydrophobic tail. The polar head of the full agonist forms a network of hydrogen bonds with the ARM-I residues (His449, His323, Ser289, Tyr327 and Tyr473) of PPARγ side chains. The hydrogen bonds formed with these residues are responsible for the conformational change of H12 and activation of PPARγ activity [31]. In contrast, partial agonists activate PPARγ by an H12 independent mechanism, wherein, the key residues in LBD are completely different to that of the full agonists in that it leads to decrease in H12 stability, thereby affecting the coactivators binding, which, in turn, reduces the transcriptional activity of PPARγ [32, 33]. Most of the previous studies suggested that partial agonists form hydrogen bond with Ser342 of LBD [31, 33].

**Fig. 1:**
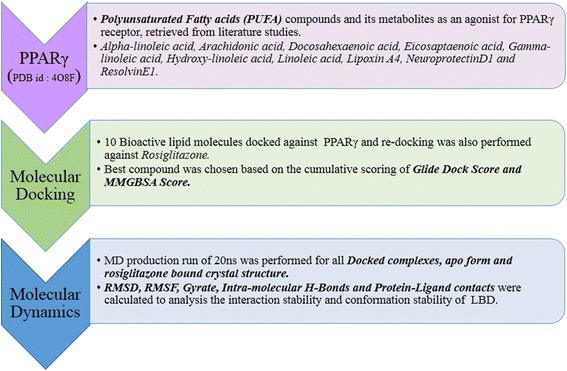
Schematic representation of in silico study on PPARγ agonists. (Note: PUFA molecule and its metabolites are collectively referred as bioactive lipids)

PPARγ agonists reduce lipid levels, enhance insulin sensitivity and thus, show anti-diabetic and anti-inflammatory actions. PPARγ, is well documented to be activated by a wide range of fatty acid molecules and their metabolites, of which PUFAs and its metabolites play a major role in exerting beneficial effects [34]. PUFAs are also reported to play significant roles in inflammatory and immune responses [35], lowering the levels of total cholesterol, Triglycerides and Low-density lipoproteins (LDL) [36, 37], in mediating apoptosis in colon cancer cells [38] and also shown to reduce the risk of early atrial fibrillation during post cardiac surgery [39]. Molecular modelling and structural bioinformatics studies serve as powerful and efficient tools for studying intermolecular interactions and dynamic behaviour of molecules in an in silico simulated conditions [40–42]. In the present study, we performed molecular docking of PPARγ against various PUFAs and its metabolites (henceforth called as bioactive lipids) [43], keeping co-crystal bound Rosiglitazone (BRL) as a reference structure (PDB ID: 4OF8). Further, the bound complexes were subjected to molecular dynamics simulation to compare the binding efficacies and to infer the agonistic binding modes of fatty acids, which are potential therapeutic molecules [44, 45], towards identification of the most potent and efficient bioactive lipid agonist targeting PPARγ (Fig. 1).

## Methods

### Protein Preparation

As a preliminary step, the crystal structure of PPARγ in complex with Rosiglitazone (PDB ID: 4O8F) at a resolution of 1.9 Å was pre-processed using Protein preparation wizard of Schrödinger suite towards optimizing the stereochemistry by assigning proper bond order, removing steric clashes, adding hydrogen atoms, fixing the disulphide bonds, missing residues, atoms and loops. Further refinement was performed by adjusting the terminal chi rotation of Asparagine, Glutamine and Histidine residues. Optimal protonation states for Histidine residues were also assigned followed by the removal of unwanted hetero groups. Finally, energy minimization was performed with OPLS2005 to obtain the optimal geometry favourable for the commencement of plausible docking studies.

### Receptor grid generation

The receptor grid was generated using the grid generation option of the Schrodinger suite across the PPARγ LBD domain to perform a targeted docking study. The grid was fixed across residues that play a critical role at the three different arms of the LBD by setting the vanderwaals radii for the receptor with a scaling factor of 1.0 Ǻ and a partial cut off of 0.25 Ǻ.

### Ligand Preparation

To expedite the protein-ligand docking workflow, all the ligand molecules were optimized using Schrodinger Ligprep module by fixing the vanderwaals radii with the scaling factor of 0.80 Ǻ and a partial charge cut-off of 0.15 Ǻ. Finally, the optimized compounds were energy minimized with OPLS 2005 as force field.

### Receptor- Ligand Docking

Docking studies establish interactions between protein and ligand molecules, thereby, aids in identifying the most favourable binding pose forming a stable complex with significant binding affinity, as scored by the docking score [46]. In this study, protein-ligand docking was performed using Schrodinger Glide version [7.0] across the grid set over the LBD region. Here, a flexible docking was performed against 10 ligand molecules, in which the best binding pose among 100 generated poses for each molecule was determined based on the Glide XP docking score. A rigid Re-docking was also performed for the rosiglitazone (BRL) against its bound crystal structure by setting the grid across the LBD so as to infer the predictive efficacy.

### MMGBSA Scoring

Molecular Mechanics Generalized Born Surface Area (MMGBSA) scoring was also performed for all the docked complexes to calculate the binding free energies by implementing the equation through Schrödinger Prime Module:

$$ \boldsymbol{\Delta} {\mathbf{G}}_{\mathbf{G}\mathbf{B}} = \boldsymbol{\Delta} {\mathbf{E}}_{\mathbf{MM}} + \boldsymbol{\Delta} {\mathbf{G}}_{\mathbf{solv}} + \boldsymbol{\Delta} {\mathbf{G}}_{\mathbf{SA}} $$

Where electrostatic solvation energy (ΔG_GB_) was calculated by using the GB Models, ΔE_MM_ is the difference between the minimized energies of ligand-protein complex and the total energies of protein and ligand in free form. ΔG_solv_ is the difference in the GBSA solvation energies of the ligand-receptor complex and the sum of the solvation energies of receptor and ligand in the unbound state. ΔG_SA_ is the difference in the surface area energies for the free receptor and the ligand. It is used to identify plausible binding conformations among the docked complexes in terms of binding free energy towards further stringent ranking of the complexes [47].

### Molecular Dynamics simulation

Molecular dynamics simulation was performed for apo, co-crystal structure (PDB id: 4O8F) and for all the docked complexes using Desmond 3.6. The system was built using a cubical box solvated with Simple Point Charge (SPC) water model. Subsequently, the system was neutralized by adding 4 Na^+^ ions for apo and 5Na^+^ ions for complexes at a concentration of ~6.22 mM. Further, this system was energy minimized with OPLS2005 as force field [48]. SHAKE algorithm was applied to restrain the geometry of water molecules, bond lengths and angles of heavy atoms and to constrain covalent bonds during MD simulation [49]. Periodic Boundary Conditions (PBC) were applied to stimulate a continuous system [50] and Particle Mesh Ewald method (PME) was applied for long range electrostatics [51]. Further, the system was equilibrated with NPT ensemble by setting temperature and pressure parameter to 300 K and 1.0 Bar, respectively. Nose-Hoover chain and Martyna-Tobias-Klein was chosen as a coupling algorithm for temperature and pressure, respectively [52, 53]. Further, the equilibrated system with a total of 48,294 atoms was simulated for 20 ns (nanosecond) with a time step of 2 fs (femtosecond) and trajectories were recorded after every 1.0 ps. The Root Mean Square Deviation (RMSD) was calculated for the backbone atoms and were graphically analysed at a time point scale [54, 55]. Similarly, root mean square fluctuation (RMSF) for each residue was also calculated to compare the major conformational changes in the residues between apo form and docked complex forms by keeping the rosiglitazone (BRL) bound crystal structure as a reference [56]. The radius of gyration was also calculated to infer the compactness of protein-ligand complexes for the comparison with apo form [57]. The 2D inter-molecular interaction plots depicting the complex stability during the MD run was also generated to infer the stability of all the complex structures.

## Results

### Comparative Molecular Docking studies of PPARγ with bioactive lipids

**Fig. 2:**
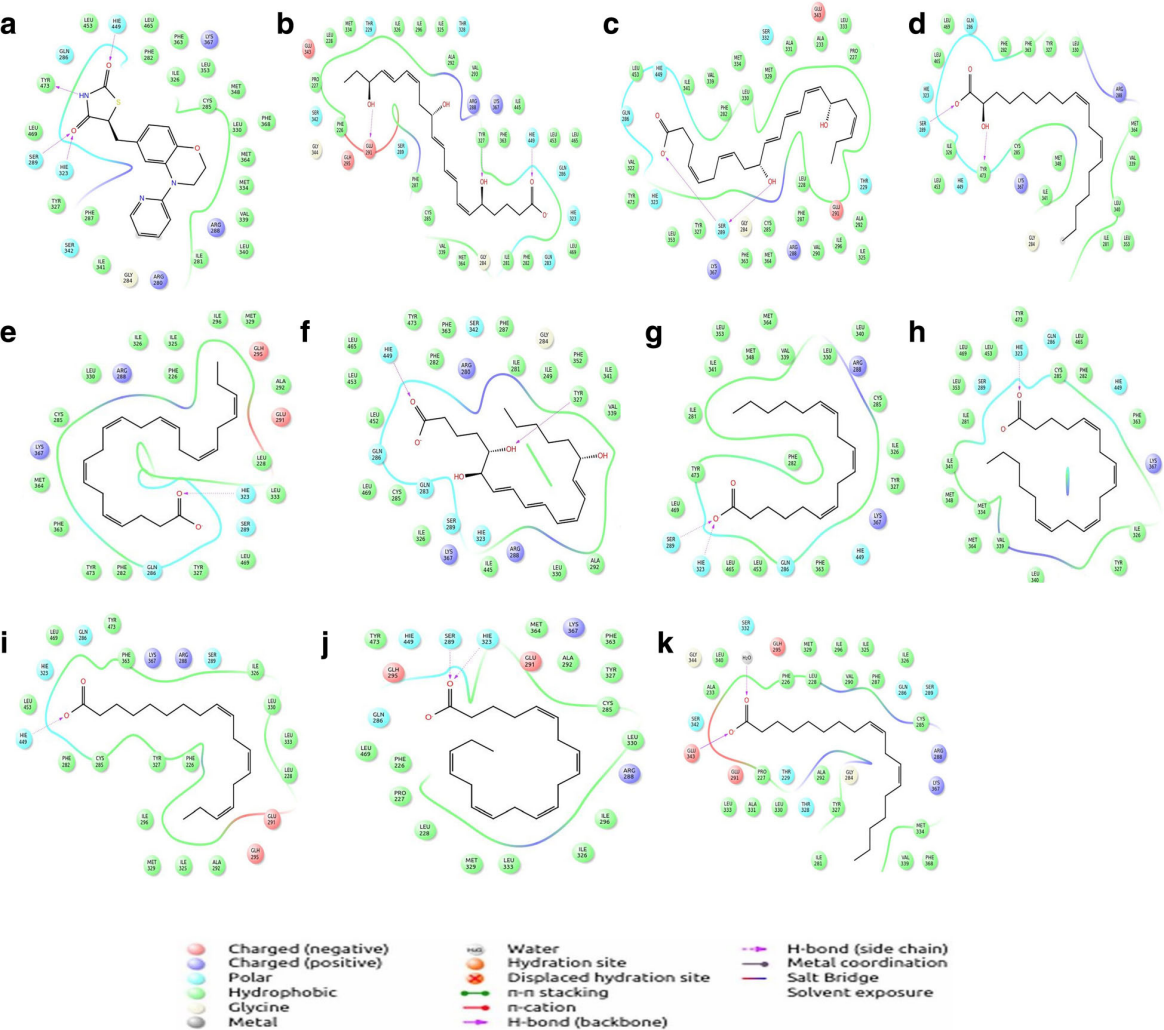
2D ligplot interaction diagram of all the 10 docked bioactive lipids complexed with PPARγ and re-docked rosiglitazone. **a** Rosiglitazone **b** Resolvin E1 **c** Neuroprotectin D1 **d** Hydroxylinoleicacid **e** Docosahexaenoicacid **f** LipoxinA4 **g** Gammalinoleicacid **h** Arachidonicacid **i** Alphalinoleicacid **j** Eicosapentaenoic acid **k** Linoleic acid

Molecular Docking was performed for all the 10 bioactive lipid compounds against the PPARγ-LBD. Glide XP dock score and MMGBSA binding free energy score were calculated for all receptor-ligand docked complexes, which revealed the scores to be in the range of −4.8 to −9.9 kcal/mol and −71.147 to −106.046 kcal/mol (Table 1), respectively. Further, 2D interaction maps with a cut-off of 4 Å for each docked complex was generated to visualize the intermolecular interactions. This inferred that all the compounds to be majorly stabilized by hydrogen bonds during complex formation (Fig. 2). The re-docking of Rosiglitazone to PPARγ-LBD also showed an agreeable deviation of 0.4 Å, inferring the predictive accuracy. The 2D maps were further scrutinized for ligand contacts with the hotspot residues spanning Arm-I, Arm-II, Arm-III and AF-2 domain towards classifying potential full and partial agonists.

**Table 1:** Receptor-Ligand docking results with Glide XP score, H-bonds and MMGBSA

Compounds	XP Glide score kcal/mol	XH-bond Interactions		MMGBSA score kcal/mol
		Donor	Acceptor	
Rosiglitazone (BRL)	−6.833	Hie449:NH	BRL: O	−105.038
		Tyr473:OH	BRL:HN	
		Ser289:OH	BRL: O	
		Hie323:NH	BRL: O	
Resolvin E1 (RsvE1)	−9.900	Hie449:NH	REV1: O	−106.046
		Tyr327:HO	REV1: HO	
		Glu291: O	REV1: HO	
Neuroprotectin D1 (NPD1)	−9.664	Ser289:OH	NPD1: O^**−**^	−102.57
		Ser289:OH	NPD1: OH	
Hydroxy-linoleic acid (H-LA)	−6.235	Tyr473:OH	HLA:HO	−95.027
		Ser289:OH	HLA: O^**−**^	
Docosahexaenoic acid (DHA)	−7.925	Hie323:NH	AA: O	−89.785
Lipoxin A4 (LXA4)	−5.796	Tyr327:OH	LXA4: HO	−87.547
		Hie449:NH	LXA4: O	
Gamma-linoleic acid (GLA)	−5.068	Hie323:NH	GLA: O^−^	−77.117
		Ser289:OH	GLA: O^−^	
Arachidonic acid (AA)	−7.040	Hie323:NH	AA: O	−76.586
Alpha-linoleic acid (AL)	−5.174	Hie449:NH	AL: O^−^	−73.657
Eicosapentaenoic acid (EPA)	−7.126	Hie323:NH	EPA: O	−71.147
		Ser289:OH	EPA: O	
Linoleic acid (LA)	−4.820	Glu343:NH	LA: O^−^	−78.026

Compounds are ranked collectively based on XP glide score and MMGBSA score showing significance with reference ligand rosiglitazone (RsvE1 > NPD1 > HLA > DHA > LXA4 > GLA > AA > ALA > EPA > LA)

### Molecular dynamics simulation of the docked complexes

An unrestrained molecular dynamics simulation study of apo form and all docked complexes were performed to infer the backbone stability, residue fluctuations, structural compactness measured in terms of RMSD, RMSF, and Rg, respectively. The RMSD trajectory revealed that all complexes to be stable during the entire production run with the system convergence at ~15 ns (Fig. 4). The RMSF trajectory inferred maximum fluctuations at L1 (238–251) and L2 (260–276) regions across all the apo and docked complexes (Fig. 5a). Rg trajectory also revealed structural compactness in apo and protein-ligand complexes (deviation within 1 Å) (Fig. 5b). A trajectory of Intra-molecular hydrogen bond counts (mean average of 210 H-bonds) was plotted for the entire production run. As expected, there was a gain in h-bonds in docked complexes in comparison with holo forms (Fig. 5c). For the top three ranking hits, Protein-ligands contact bar charts were plotted, which inferred the contribution of hotspot residues in establishment of intermolecular contacts, and was majorly found to be h-bond mediated (Fig. 6).

## Discussion

### Receptor-Ligand Docking analysis

Molecular Docking studies were subsequently performed for all the bioactive lipids against the assigned grid surface on the protein. The docked complexes were analysed for the Glide XP score, MMGBSA score and H-bond interactions, to collectively infer the ligand binding affinity [58, 59]. The Glide receptor-ligand docking results are tabulated in (Table 1). Among the docked complexes, Resolvin E1 (RsvE1), neuroprotectin D1 (NPD1) and hydroxy-linoleic acid (H-LA) showed significant affinity compared to other compounds in terms of XP dock score of −9.900 kcal/mol, −9.664 kcal/mol and −6.235 kcal/mol, along with a relative MMGBSA score of −106.046 kcal/mol, −102.57 kcal/mol and −95.027 kcal/mol, respectively. RsvE1 displayed strong intermolecular interactions, by forming three H-bonds with the key side chain amino acids of LBD: amino group of Hie449 (H11), hydroxyl group of Tyr327 and Oxyl group of Glu291. NPD1 showed two H-bonds with the side chain of Ser289 and H-LA formed two H-bond interactions with the OH group of side chain amino acids Tyr473 (H12) and Ser289 (H3), respectively. Moreover, RsvE1, NPD1 and H-LA also showed a significant scoring comparable to that of the reference ligand rosiglitazone (BRL), both in terms of docking score as well as MMGBSA score. Rosiglitazone on re-docking with the crystal structure yielded a XP dock score of −6.833 kcal/mol and MMGBSA score of −105.038 kcal/mol. The glide re-docked complex of rosiglitazone (BRL) showed a RMSD of 0.446 Ǻ on structural alignment with the native PPARγ-Rosiglitazone co-crystallized structure, inferring the predictive accuracy of the method implemented (Table 1).

RsvE1, NPD1 and H-LA formed hydrogen bonds with the arm-I residues Hie449 (H11), Ser289(H3) and Tyr327, which are reported to be important for producing full activity of the compound by direct stabilization of H12 helix and are responsible for the transactivation activity of PPARγ [24, 31, 60, 61]. HLA also formed a hydrogen bond with Tyr473 (H12) located on the arm I that harbours transcription activation function-2(AF-2), which is obligatory for ligand binding and as well as in complimenting PPARγ function [27]. Tyr473 is also hypothesized to play a crucial role in AF-2 stabilization, as it occupies helix12 which closes the ligand binding site upon ligand binding. This activity of helix12 favours in the reduction of conformational fluctuations that sets an optimal LBD structure for co-activator binding. It has also been reported that mutation at Tyr473 leading to the loss of agonistic activation by the ligands.

Alpha-linoleic acid (ALA) showed a Glide XP dock score of −5.174 kcal/mol by forming a single H-bond with the side chain of Hie449 (H11). Arachidonic acid (AA) and docosahexaenoic acid (DHA) formed a single H-bond with the side chain of Hie323 with an XP dock score of −7.040 kcal/mol and −7.925 kcal/mol, respectively. Eicosapentaenoic acid (EPA) and gamma-linoleic acid (GLA) displayed a XP dock score of −7.126 kcal/mol and −5.068 kcal/mol by forming two hydrogen bonds with the side chain residues of Hie323 and Ser289, respectively. Lipoxin A4 (LXA4) with a docking XP score of −5.796 kcal/mol, displayed two hydrogen bond interactions with the active site residues Hie 449 and Tyr327. Linoleic acid (LA) showed the least XP dock score of −4.820 kcal/mol among all the docked bioactive lipids compounds by forming an H-bond with the back bone of Glu342 (Table 1).

**Fig. 3:**
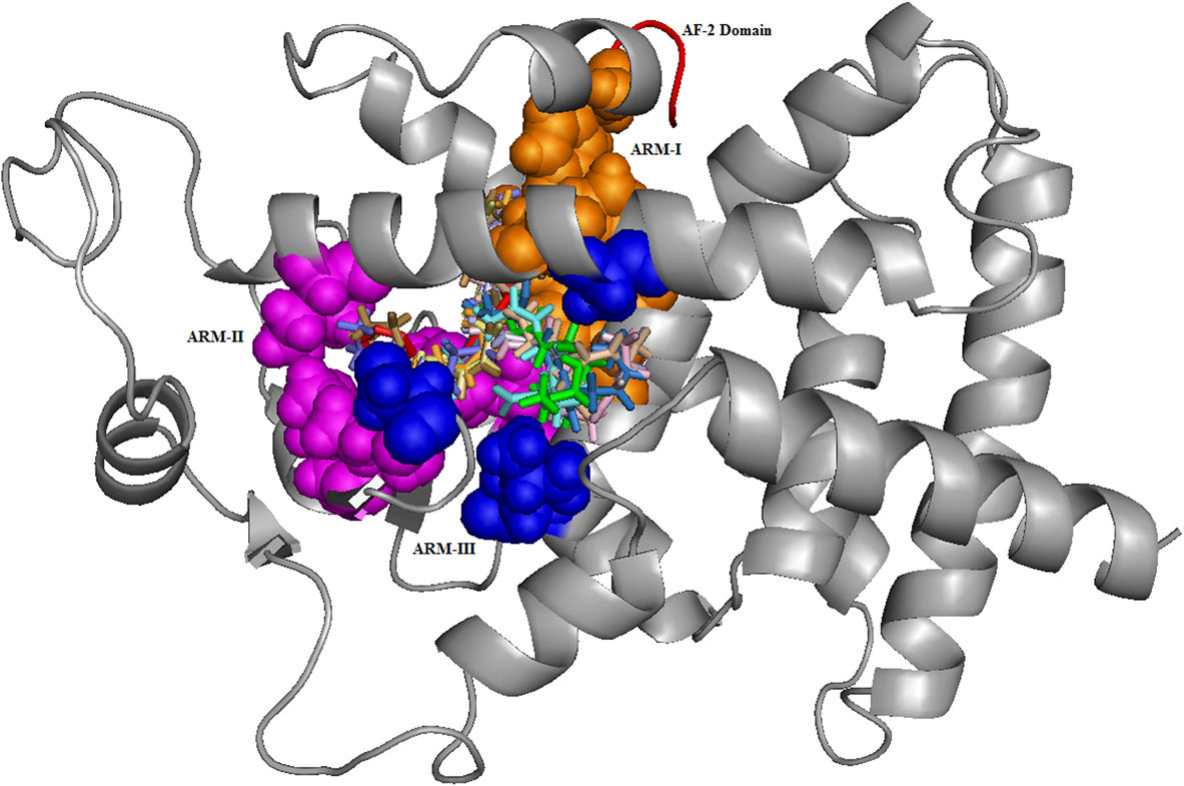
All the 10 bioactive lipids were found to be docked within the three arms of the Ligand Binding Domain (LBD). All the ligands are represented as sticks with different colours (BRL: red, RsvE1: green, NPD1: sky blue, H-LA: pale green, DHA: light blue, LXA4: sand, GLA: orange, AA: pale yellow, AL: wheat, EPA: light pink, LA: aquamarine). ARM I (orange) involving four key residues (Tyr^473^, His^323^, His^449^, Ser^289^ and Tyr^327^) and AF-2 at the C-terminal end of LBD critical for co-activator binding pocket. ARM II (magenta) constitutes six residues (Met^364^, Ile^281^, Met^348^, Ile^341^ and Lys^367^) and ARM III (blue) with (Ala^292^, Leu^333^ & Ser^342^)

The ligand interactions with ARM-I side chains of Hie323 (H3), Hie449 (H11), Ser289(H3) and Tyr327 of LBD is reported to play a significant role in enhancing agonistic activity as it is found to indirectly influence the H12 stabilization [24, 31, 60, 61]. Of the 10 docked complexes, 9 bioactive lipids are predicted to act as full agonists for PPARγ by forming an H-bond interaction with the ARM-I hydrophilic cavity residues (Hie 323, Hie 449, Tyr473, Ser289 and Tyr327). Linoleicacid has been found to be a partial agonist for PPARγ as it formed H-bond interactions with Glu343 of ARM-III [24]. The superimposition of all ten ligand molecules (bioactive lipids studied in the present study) along with the rosiglitazone as a reference ligand, inferred all the ligands to be well bound within the three arms of agonist binding domain by interacting with key residues (Figs. 2 and 3) [32, 62].

### Molecular dynamics studies of the protein-ligand complexes

**Fig. 4:**
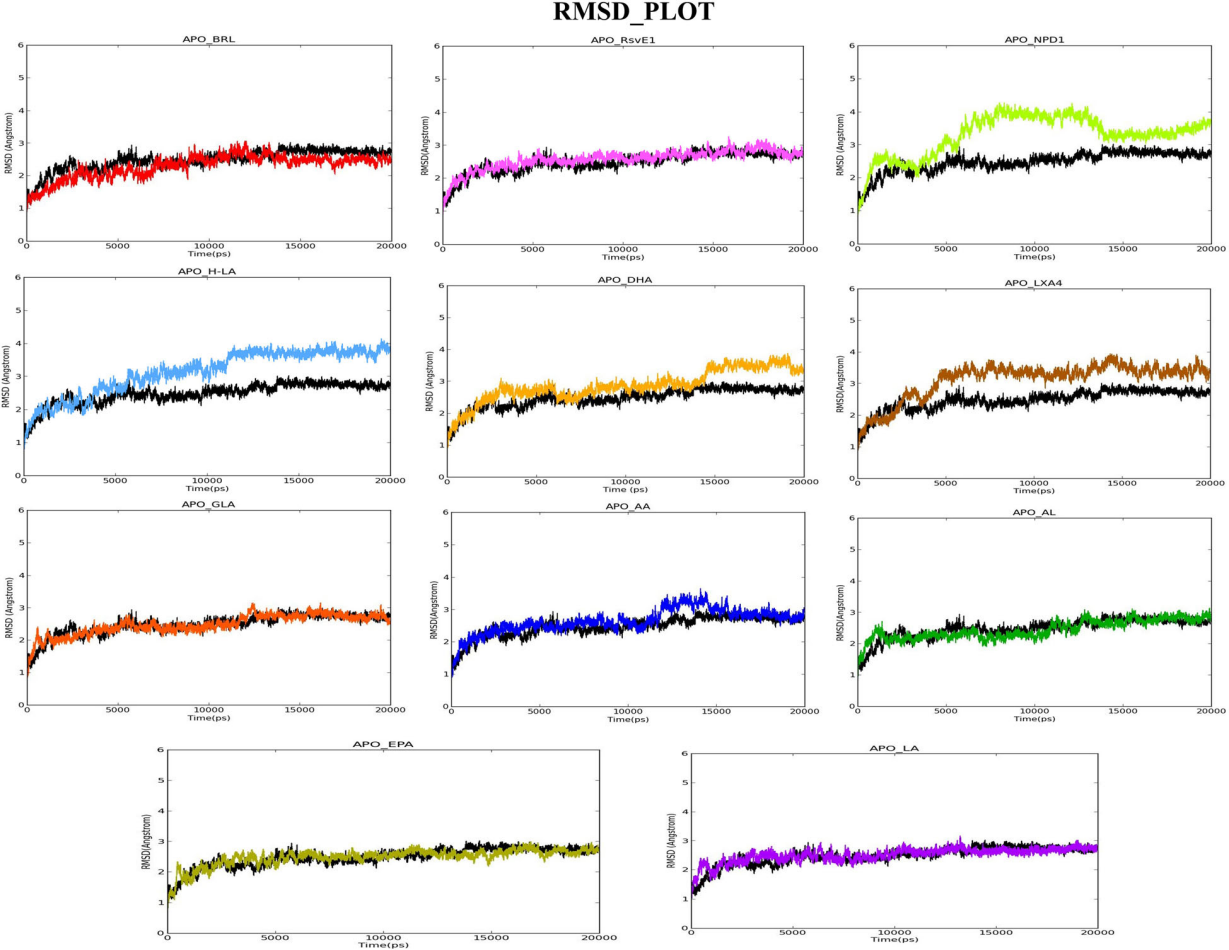
RMSD graph showing the backbone atoms convergence of all the complex structures

**Fig. 5:**
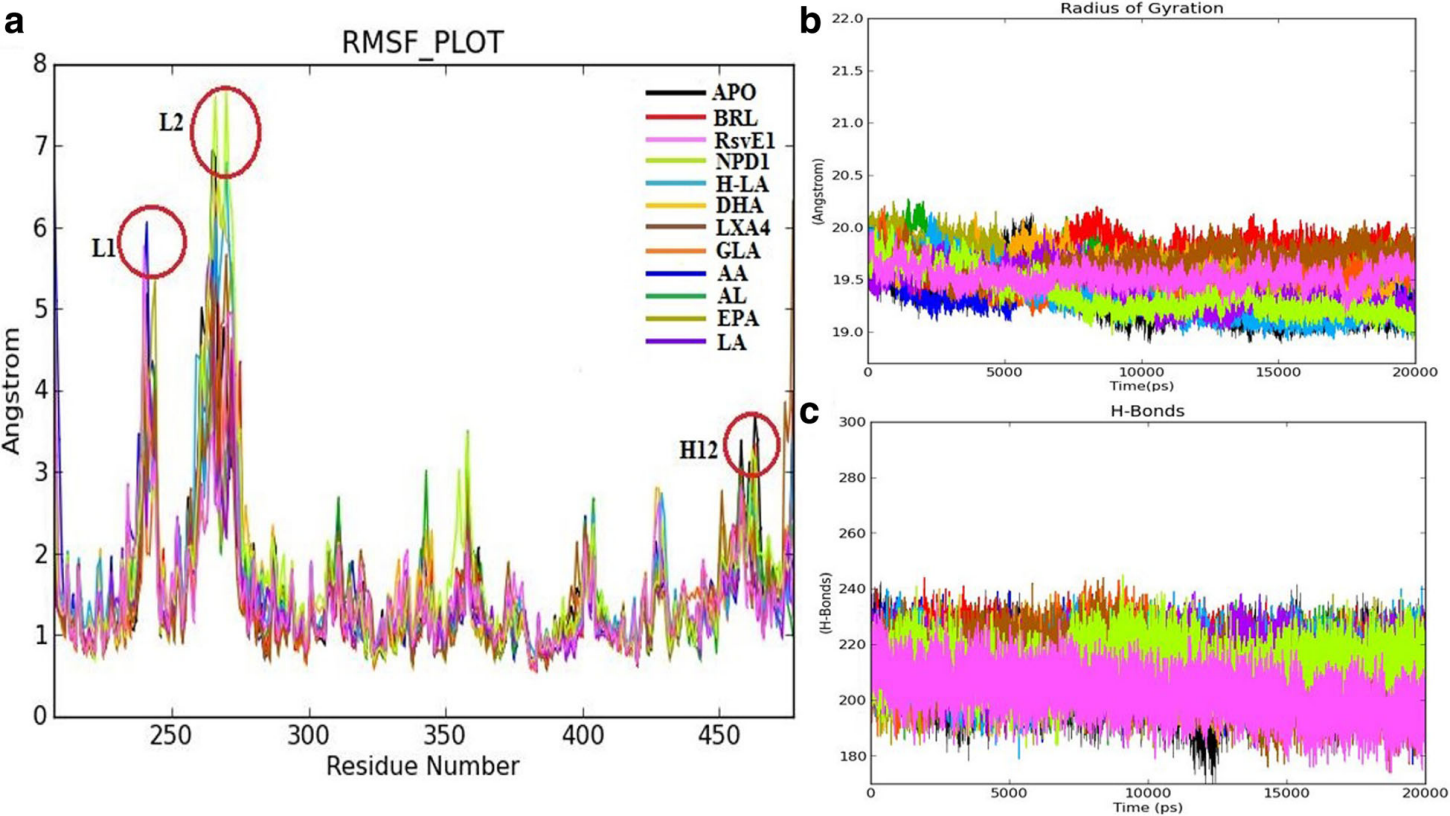
**a** RMSF graph displaying major fluctuations at the two lengthy loop segments L1 and L2 and Helix 12 (H12) in all the holo forms showing a less fluctuation than the apo form. **b** Radius of gyration graph staging the compactness of the protein-ligand complexes and **c** Intra-molecular H-bonds showcasing no major loss in the protein complex secondary structures

**Fig. 6:**
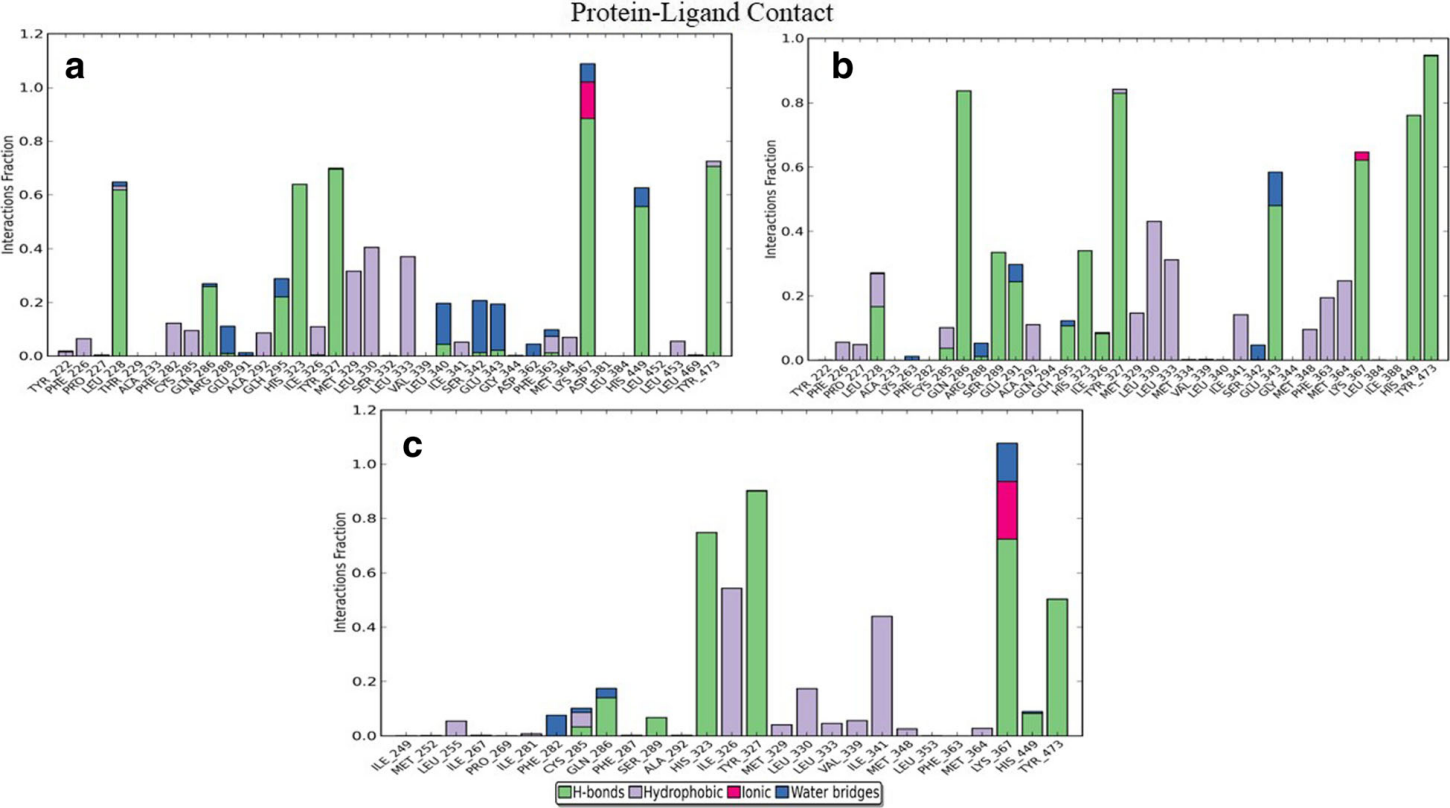
**a-c** Protein-ligand contact of RsvE1, NDP1 and H-LA with PPARγ showing strong intermolecular interactions with the key active site residues over the period of simulation 20 ns

The RMSD trajectory of the backbone atoms of apo form and all the complex structures showed a convergence after a time frame ranging from ~ 13,000 ps to ~ 15,000 ps with a maximum mean value of ~3.5Ǻ and S.D of 0.675Ǻ (Fig. 4). The RMSF fluctuation has reached the maximum peak limit of 6-7Ǻ at the residues residing over the lengthy loop region (Loop1 with 14 residues and Loop2 with 17 residues) and remaining residues showed fluctuations within 1Ǻ. The H12 (Tyr473) residue showed a slightly higher fluctuation in the *apo* form compared to all the other protein-ligand complexes. This corroborates with the hypothesis that upon ligand binding to H12 hotspot to confer closure of LBD, thereby stabilizes the LBD conformation in the ligand bound state (Fig. 5a) [27]. The *apo* form and all the 10 (bioactive lipids) complexes were found to be compact; by displaying a radius of gyration less than ~ 1.3 Ǻ (Fig. 5b). The intra-molecular H-bond graph also inferred no major loss in the protein secondary structure conformations, thereby, reinforcing that the protein-ligand complex structures to be well stabilized (Fig. 5c). However, the protein complexed with the ligands Rsv E1, NPD1 and H-LA were found to establish strong protein-ligand contacts by constituting plausible intermolecular H-bonds with the active site residues of the LBD domain throughout the period of simulation. RsvE1, NPD1 and H-LA displayed a consistent and plausible H-bond interactions with the critical residues of ARM-1 (Hie323, Tyr327, Hie449, Lys367 and Tyr473) for ~50% during the production run (Fig. 6a-c). The three bioactive lipids (RsvE1, NDP1 and H-LA) interacting with two conserved side chain amino acids Tyr327 and Lys367, are reported for favouring the formation of H-bonds at the Keto-group of the ligands. These amino acid side chains are known to play a vital role in determining the specificity of ligands that can efficiently couple with the receptor [62] (Fig. 6a and b). It is noteworthy that these residues of arm-1 (His323, His449, Tyr473, Tyr327 and Lys367) are hypothesized for indirect and direct stabilisation of H12 (Tyr473), thereby favouring for co-activator binding and for contributing towards the transactivity of PPARγ.

## Conclusion

In the present study, we performed a molecular docking analysis with bioactive lipid compounds which are documented to be agonists of PPARγ [30]. All the 10 docked complexes were found to be well bound within the three different arms of LDB. Thereby in-order to perform a further comparative study on the protein-ligand interaction stability and the conformational stability of the LBD, molecular dynamics studies were carried out for apo form, LBD co-crystallized with rosiglitazone and for all the docked complexes with bioactive lipids.

The cumulative analysis based on the Glide score, Prime-MMGBSA free binding energy score and MD trajectories analysis infer that RsvE1, NDP1 and H-LA to be the best docked compounds as these three showed relatively a significant score to that of the re-docking score of the reference ligand Rosiglitazone in terms of glide XP score, MMGBSA score and H-bond forming pattern with the key residues (Table 1). The MD trajectory also reinforce that RsvE1, NDP1 and H-LA to be the best compounds based upon the RMSF graph (Fig. 5a), which clearly depicts that upon RsvE1, NDP1 and H-LA ligands binding to the LBD, it displays a much lesser fluctuation at H12 region in comparison to that of the reference ligand Rosiglitazone (BRL) and thereby maintaining a stable LBD structure for co-activator binding.

The protein-ligand contacts of the RsvE1, NDP1 and H-LA with PPARγ throughout the production run of 20 ns also corroborates well by displaying a strong intermolecular interaction with the key active site residues of the LBD domain over ~50% of the simulation time, which confirms the stability upon the ligand binding and its intactness with the receptor.

Moreover, RsvE1 is found to mimic the insulin sensitizing and anti-steatotic activities of omega-3-PUFAs, and also found to induce adiponectin expression similar to rosiglitazone [63]. NPD1 interacting with the PPARγ is hypothesized to be play a significant role in suppressing neuroinflammation and thus, is considered to be of benefit in some neurodegenerative diseases [64]. Hydroxy-linoleic acid (H-LA) is reported to have anti-proliferative action on tumor cells [65]. In the present study, we have ranked all these PUFA compounds collectively based on the Glide docking score, H-bonding formation with the key residues, Prime MMGBSA score and MD simulation trajectory analyses and observed that their relative PPARγ agonist activity to be as follows: RsvE1 > NPD1 > H-LA > DHA > LXA4 > GLA > AA > ALA > EPA > LA. However, Further experimental studies need to be performed to verify the results obtained in the present bioinformatics analysis. Such studies could include the assessment of ability of these bioactive lipids in the pathobiology of obesity, insulin resistance, type 2 DM, metabolic syndromes and cancer.

## Abbreviations

AA: Arachidonic acid
AF-2: Transcription Activation Function-2
AL: Alpha-linoleic acid
BRL: Rosiglitazone
DHA: Docosahexaenoic acid
EPA: Eicosapentaenoic acid
GLA: Gamma-linoleic acid
H-LA: Hydroxy-linoleic acid
LA: Linoleic acid
LBD: Ligand Binding Domain
LDL: Low density lipoprotein
LXA4: Lipoxin A4
MD: Molecular dynamics
MMGBSA: Molecular Mechanics Generalized Born Surface Area
NPD1: Neuroprotectin D1
OPLS: Optimized Potentials for Liquid Simulations
PBC: Periodic boundary conditions
PME: Particle Mesh Ewald
PPARα: Peroxisome proliferator-activated receptor alpha
PPARβ: Peroxisome proliferator-activated receptor beta
PPARγ: Peroxisome proliferator-activated receptor gamma
PUFAs: Poly unsaturated fatty acids
Rg: Radius of gyration
RMSD: Root mean square deviation
RMSF: Root mean square fluctuation
RsvE1: Resolvin E1
SPC: Simple point charge
XP: Extra precision score

## References

[1] Guasch L, Sala E, Castell-Auvi A, Cedo L, Liedl KR, Wolber G (2012). Identification of PPARgamma partial agonists of natural origin (I): development of a virtual screening procedure and in vitro validation. PLoS One.

[2] Grygiel-Gorniak B (2014). Peroxisome proliferator-activated receptors and their ligands: nutritional and clinical implications--a review. Nutr J.

[3] Willson TM, Brown PJ, Sternbach DD, Henke BR (2000). The PPARs: from orphan receptors to drug discovery †. J Med Chem.

[4] Berger J, Moller DE (2002). The mechanisms of action of PPARs. Annu Rev Med.

[5] Yu S, Reddy JK (1771). Transcription coactivators for peroxisome proliferator-activated receptors. Biochim Biophys Acta.

[6] Feige JN, Auwerx J (2007). Transcriptional coregulators in the control of energy homeostasis. Trends Cell Biol.

[7] Wang L, Waltenberger B, Pferschy-Wenzig E, Blunder M, Liu X, Malainer C (2014). Natural product agonists of peroxisome proliferator-activated receptor gamma (PPARgamma): a review. Biochem Pharmacol.

[8] Sertznig P, Seifert M, Tilgen W, Reichrath J (2007). Present concepts and future outlook: function of peroxisome proliferator-activated receptors (PPARs) for pathogenesis, progression, and therapy of cancer. J Cell Physiol.

[9] Neschen S, Morino K, Dong J, Wang-Fischer Y, Cline GW, Romanelli AJ (2007). n-3 Fatty acids preserve insulin sensitivity in vivo in a peroxisome proliferator-activated receptor-alpha-dependent manner. Diabetes.

[10] Delerive P, Furman C, Teissier E, Fruchart J, Duriez P, Staels B (2000). Oxidized phospholipids activate PPARalpha in a phospholipase A2-dependent manner. FEBS Lett.

[11] Kliewer SA, Sundseth SS, Jones SA, Brown PJ, Wisely GB, Koble CS (1997). Fatty acids and eicosanoids regulate gene expression through direct interactions with peroxisome proliferator-activated receptors alpha and gamma. Proc Natl Acad Sci U S A.

[12] Lo Verme J, Fu J, Astarita G, La Rana G, Russo R, Calignano A, Piomelli D (2005). The nuclear receptor peroxisome proliferator-activated receptor-alpha mediates the anti-inflammatory actions of palmitoylethanolamide. Mol Pharmacol.

[13] Sheu S, Kaya T, Waxman DJ, Vajda S (2005). Exploring the binding site structure of the PPAR gamma ligand-binding domain by computational solvent mapping. Biochemistry.

[14] Medina-Gomez G, Gray SL, Yetukuri L, Shimomura K, Virtue S, Campbell M (2007). PPAR gamma 2 prevents lipotoxicity by controlling adipose tissue expandability and peripheral lipid metabolism. PLoS Genet.

[15] Wang Y, Lee C, Tiep S, Yu RT, Ham J, Kang H, Evans RM (2003). Peroxisome-proliferator-activated receptor delta activates fat metabolism to prevent obesity. Cell.

[16] Stephen RL, Gustafsson MCU, Jarvis M, Tatoud R, Marshall BR, Knight D (2004). Activation of peroxisome proliferator-activated receptor delta stimulates the proliferation of human breast and prostate cancer cell lines. Cancer Res.

[17] Jialal I, Smith G (2012). Managing the dyslipidemia of metabolic syndrome: beyond statin therapy. Metab Syndr Relat Disord.

[18] Wang Y, Jacome-Sosa MM, Ruth MR, Lu Y, Shen J, Reaney MJ (2012). The intestinal bioavailability of vaccenic acid and activation of peroxisome proliferator-activated receptor-alpha and -gamma in a rodent model of dyslipidemia and the metabolic syndrome. Mol Nutr Food Res.

[19] Wilding JPH (2012). PPAR agonists for the treatment of cardiovascular disease in patients with diabetes. Diabetes Obes Metab.

[20] Wayman NS, Hattori Y, McDonald MC, Mota-Filipe H, Cuzzocrea S, Pisano B (2002). Ligands of the peroxisome proliferator-activated receptors (PPAR-gamma and PPAR-alpha) reduce myocardial infarct size. FASEB J.

[21] Bolden A, Bernard L, Jones D, Akinyeke T, Stewart LV (2012). The PPAR gamma agonist troglitazone regulates Erk 1/2 phosphorylation via a PPARgamma-Independent, MEK-dependent pathway in human prostate cancer cells. PPAR Res.

[22] Robbins GT, Nie D (2012). PPAR gamma, bioactive lipids, and cancer progression. Front Biosci (Landmark Ed).

[23] Segawa Y, Yoshimura R, Hase T, Nakatani T, Wada S, Kawahito Y (2002). Expression of peroxisome proliferator-activated receptor (PPAR) in human prostate cancer. Prostate.

[24] Guasch L, Sala E, Valls C, Blay M, Mulero M, Arola L (2011). Structural insights for the design of new PPARgamma partial agonists with high binding affinity and low transactivation activity. J Comput Aided Mol Des.

[25] Fyffe SA, Alphey MS, Buetow L, Smith TK, Ferguson MAJ, Sorensen MD (2006). Recombinant human PPAR-beta/delta ligand-binding domain is locked in an activated conformation by endogenous fatty acids. J Mol Biol.

[26] Markt P, Schuster D, Kirchmair J, Laggner C, Langer T (2007). Pharmacophore modeling and parallel screening for PPAR ligands. J Comput Aided Mol Des.

[27] Zoete V, Grosdidier A, Michielin O (1771). Peroxisome proliferator-activated receptor structures: ligand specificity, molecular switch and interactions with regulators. Biochim Biophys Acta.

[28] Gurula H, Loganathan T, Krishnamoorthy T, Vetrivel U, Samuel S (2015). Virtual screening studies of seaweed metabolites for predicting PPARγ agonists. Int J Pharm Pharmaceutical Sci.

[29] Xu HE, Lambert MH, Montana VG, Parks DJ, Blanchard SG, Brown PJ (1999). Molecular recognition of fatty acids by peroxisome proliferator-activated receptors. Mol Cell.

[30] Sundriyal S, Bharatam PV (2009). Important pharmacophoric features of pan PPAR agonists: common chemical feature analysis and virtual screening. Eur J Med Chem.

[31] Farce A, Renault N, Chavatte P (2009). Structural insight into PPARgamma ligands binding. Curr Med Chem.

[32] Bruning JB, Chalmers MJ, Prasad S, Busby SA, Kamenecka TM, He Y (2007). Partial agonists activate PPARgamma using a helix 12 independent mechanism. Structure.

[33] Lu I, Huang C, Peng Y, Lin Y, Hsieh H, Chen C (2006). Structure-based drug design of a novel family of PPARgamma partial agonists: virtual screening, X-ray crystallography, and in vitro/in vivo biological activities. J Med Chem.

[34] El Akoum S. PPAR Gamma at the Crossroads of Health and Disease: A Masterchef in Metabolic Homeostasis. Endocrinol Metab Synd. 2014. doi:10.4172/2161-1017.1000126.

[35] Bhagat U, Das UN (2015). Potential role of dietary lipids in the prophylaxis of some clinical conditions. Arch Med Sci.

[36] Sahebkar A, Serban M, Gluba-Brzozka A, Mikhailidis DP, Cicero AF, Rysz J, Banach M (2016). Lipid-modifying effects of nutraceuticals: An evidence-based approach. Nutrition.

[37] Banach M, Aronow WS, Serban M, Rysz J, Voroneanu L, Covic A (2015). Lipids, blood pressure and kidney update 2015. Lipids Health Dis.

[38] Zhang C, Yu H, Shen Y, Ni X, Shen S, Das UN (2015). Polyunsaturated fatty acids trigger apoptosis of colon cancer cells through a mitochondrial pathway. Arch Med Sci.

[39] Mariscalco G, Sarzi Braga S, Banach M, Borsani P, Bruno VD, Napoleone M (2010). Preoperative n-3 polyunsatured fatty acids are associated with a decrease in the incidence of early atrial fibrillation following cardiac surgery. Angiology.

[40] Umashankar V, Gurunathan S, Ballantyne B, Marrs TC, Syversen T, Casciano DA, Sahu SC (2009). In Silico Tools for Molecular Modeling. General, Applied and Systems Toxicology.

[41] Umashankar V, Gurunathan S, Ballantyne B, Marrs TC, Syversen T, Casciano DA, Sahu SC (2009). Chemoinformatics and its Applications. General, Applied and Systems Toxicology.

[42] Umashankar VGS (2015). DRUG DISCOVERY: AN APPRAISAL. Int J Pharm Pharmaceutical Sci.

[43] Vetrivel U, Ravichandran SB, Kuppan K, Mohanlal J, Das UN, Narayanasamy A (2012). Agonistic effect of polyunsaturated fatty acids (PUFAs) and its metabolites on brain-derived neurotrophic factor (BDNF) through molecular docking simulation. Lipids Health Dis.

[44] Ortuno Sahagun D, Marquez-Aguirre AL, Quintero-Fabian S, Lopez-Roa RI, Rojas-Mayorquin AE (2012). Modulation of PPAR-gamma by Nutraceutics as Complementary Treatment for Obesity-Related Disorders and Inflammatory Diseases. PPAR Res.

[45] Serhan CN, Yacoubian S, Yang R (2008). Anti-inflammatory and proresolving lipid mediators. Annu Rev Pathol.

[46] Sandeep S, Priyadarshini V, Pradhan D, Munikumar M, Umamaheswari A. Docking and molecular dynamics simulations studies of human protein kinase catalytic subunit alpha with antagonist. J Clin Sci Res. 2012:15–23 doi:10.15380/2277-5706.JCSR.12.005

[47] Genheden S, Ryde U (2015). The MM/PBSA and MM/GBSA methods to estimate ligand-binding affinities. Expert Opin Drug Discov.

[48] DuBay KH, Hall ML, Hughes TF, Wu C, Reichman DR, Friesner RA (2012). Accurate force field development for modeling conjugated polymers. J Chem Theory Comput.

[49] Barth E, Kuczera K, Leimkuhler B, Skeel RD (1995). Algorithms for constrained molecular dynamics. J Comput Chem.

[50] Bulatov VV, Rhee M, Cai W (2000). Periodic boundary conditions for dislocation dynamics simulations in three dimensions. Mater Res Soc Proc.

[51] Harvey MJ, de Fabritiis G (2009). An implementation of the smooth particle mesh ewald method on GPU hardware. J Chem Theory Comput.

[52] Kleinerman DS, Czaplewski C, Liwo A, Scheraga HA (2008). Implementations of Nose-Hoover and Nose-Poincare thermostats in mesoscopic dynamic simulations with the united-residue model of a polypeptide chain. J Chem Phys.

[53] Martyna GJ, Tobias DJ, Klein ML (1994). Constant pressure molecular dynamics algorithms. J Chem Phys.

[54] Damm KL, Carlson HA (2006). Gaussian-weighted RMSD superposition of proteins: a structural comparison for flexible proteins and predicted protein structures. Biophys J.

[55] Maiorov VN, Crippen GM (1994). Significance of root-mean-square deviation in comparing three-dimensional structures of globular proteins. J Mol Biol.

[56] Fuglebakk E, Echave J, Reuter N (2012). Measuring and comparing structural fluctuation patterns in large protein datasets. Bioinformatics.

[57] Lobanov MI, Bogatyreva NS, Galzitskaia OV (2008). Radius of gyration is indicator of compactness of protein structure. Mol Biol (Mosk).

[58] Vetrivel U, Muralikumar S, Mahalakshmi B, Lily Therese K, Madhavan HN, Alameen M, Thirumudi I (2016). Multilevel precision-based rational design of chemical inhibitors targeting the hydrophobic cleft of toxoplasma gondii Apical Membrane Antigen 1 (AMA1). Genomics Inform.

[59] John A, Sivashanmugam M, Umashankar V, Natarajan SK. Virtual screening, molecular dynamics, and binding free energy calculations on human carbonic anhydrase IX catalytic domain for deciphering potential leads. J Biomol Struct Dyn. 2016:1–14. doi:10.1080/07391102.2016.1207565. 10.1080/07391102.2016.1207565 27373313

[60] Shashni B, Sharma K, Singh R, Sakharkar KR, Dhillon SK, Nagasaki Y, Sakharkar MK (2013). Coffee component hydroxyl hydroquinone (HHQ) as a putative ligand for PPAR gamma and implications in breast cancer. BMC Genomics.

[61] Itoh T, Fairall L, Amin K, Inaba Y, Szanto A, Balint BL (2008). Structural basis for the activation of PPARgamma by oxidized fatty acids. Nat Struct Mol Biol.

[62] Tsukahara T, Tsukahara R, Yasuda S, Makarova N, Valentine WJ, Allison P (2006). Different residues mediate recognition of 1-O-oleyllysophosphatidic acid and rosiglitazone in the ligand binding domain of peroxisome proliferator-activated receptor gamma. J Biol Chem.

[63] Gonzalez-Periz A, Horrillo R, Ferre N, Gronert K, Dong B, Moran-Salvador E (2009). Obesity-induced insulin resistance and hepatic steatosis are alleviated by omega-3 fatty acids: a role for resolvins and protectins. FASEB J.

[64] Palacios-Pelaez R, Lukiw WJ, Bazan NG (2010). Omega-3 essential fatty acids modulate initiation and progression of neurodegenerative disease. Mol Neurobiol.

[65] Erazo T, Lorente M, Lopez-Plana A, Munoz-Guardiola P, Fernandez-Nogueira P, Garcia-Martinez JA (2016). The new antitumor drug ABTL0812 inhibits the Akt/mTORC1 axis by upregulating tribbles-3 pseudokinase. Clin Cancer Res.

